# Motion, Contact, or Both: Three Paths Convey Whisker Sensation in the Rat

**DOI:** 10.1371/journal.pbio.0040153

**Published:** 2006-04-18

**Authors:** Richard Robinson

In the rat, whiskers are highly specialized sensory organs that serve much the same function as eyes and hands do for humans—they are a prime source of tactile and spatial information. The study of whisker sensory processing has proven fruitful for understanding how information is received, delivered, processed, and acted on in the brain. In this issue, Chunxiu Yu, Ehud Ahissar, and colleagues present novel insights into the connection between brain structure and sensory function by showing that whisker sensations that enter the thalamus, a central gateway of the brain, travel by three distinct pathways—two of which convey signals representing specific aspects of the sensory experience, while the third conveys a complex signal.

It has been shown previously that these three pathways, called the paralemniscal, extralemniscal, and lemniscal, carry whisker sensations from sensory neurons via the thalamus and on to higher sensory-processing centers of the brain, but how these pathways handled the different types of information was unclear.

To examine this, the authors stimulated the facial nerve in anesthetized rats, causing the whisker to move as it does when the rats are actively moving their whiskers to explore the environment, a behavior known as “whisking.” Sometimes the whiskers contacted a rod placed in its path, while other times they contacted nothing. To see if the whiskers convey a different message depending on whether the rat is whisking versus when objects passively come into contact with the whisker, the authors also brought the rod in contact with stationary whiskers.

Using single-cell recording electrodes implanted in different sections of the thalamus, the authors could compare the signals sent by the sensory neurons under these various conditions. They found that whisker movement induced activity in the paralemniscal pathway, whether or not the whisker touched the rod. Contact with the rod induced activity in the extralemniscal pathway, whether or not the whisker moved. And when the moving whisker contacted the rod, both pathways were active, along with the third pathway, the lemniscal.

The authors propose that the thalamic pathways function somewhat in parallel, each specialized for handling unique dimensions of movement and touch. In this arrangement, the paralemniscal handles temporal information related to motor control of whisking, the extralemniscal conveys object location, and the lemniscal pathway integrates a higher dimension of temporal and spatial information. The authors note that each of these pathways conveys information back to the motor nuclei by a different route, and thus is involved in a unique motor-sensory-motor loop. The authors caution, however, that these loops would not function in isolation, but, instead, can be considered parallel loops, with the higher processing loops building on the lower ones.

These results strengthen a model of the nervous system in which each sensory-motor pathway evolved in steps over time, with each new addition reaching to higher brain regions and subserving novel behaviors. In this scheme, evolution of movement sensation of the whiskers, conveyed by the paralemniscal pathway and processed in low brain regions, would have arisen first. This would be followed by evolution of contact detection, conveyed by the extralemniscal pathway and processed higher up in the brain to analyze object location. Finally, as analysis of object identity required greater detail, the lemniscal pathway would arise to convey the integrated information for higher brain analysis. Further testing of this model of nested motor-sensory-motor loops in this and other sensory systems may help determine the principles of active sensation.

## 

**Figure:**
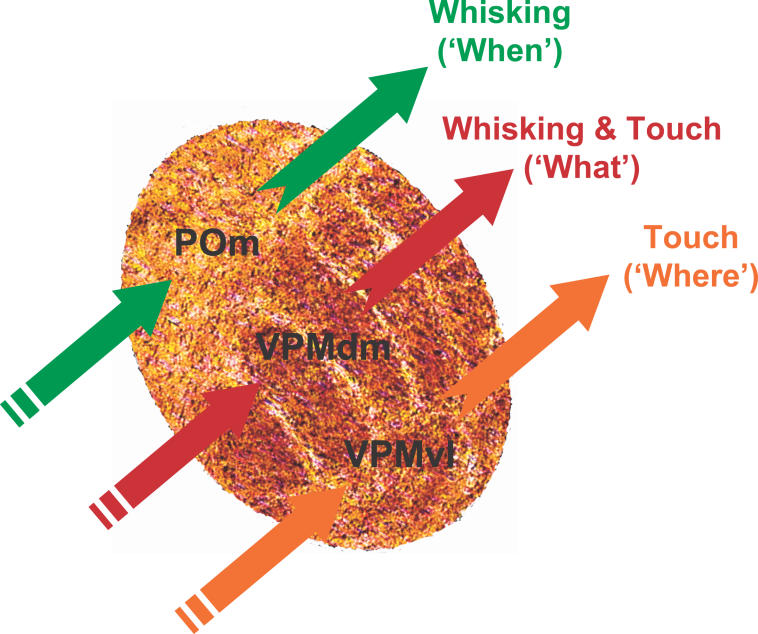
Three parallel trigeminal pathways through the thalamus convey whisking, contact, and whisking-touch signals, enabling parallel processing of sensor motion (“when”), object location (“where”), and object identity (“what”) in the whisking rat.

